# Trust as a catalyst: revealing the impact of government trust and professional trust on public health policy compliance during a pandemic

**DOI:** 10.1186/s12889-024-18449-2

**Published:** 2024-04-04

**Authors:** Guobang Chen, Hua Zhang, Yue Hu, Chunyan Luo

**Affiliations:** School of Political Science and Public Administration, Guangxi Minzu University, Nanning, Guangxi China

**Keywords:** Government trust, Professional trust, Compliance behaviour, Pandemic

## Abstract

**Background:**

Existing research has extensively explored the relationship between government trust and compliance behaviour, but significant controversies exist. Some studies suggest a strong positive correlation between the two. Other studies have found that government trust hinders compliance behaviour. However, during the pandemic, the effectiveness of public health policies largely depends on the public’s compliance with these policies. To examine the aforementioned controversies, this study utilizes survey data on the Chinese population during the COVID-19 period to explore the relationship between compliance with public health policies and government trust.

**Methods:**

The study conducted a questionnaire survey of 1,395 individuals from 25 provinces in China from mid-November to mid-December 2022. Firstly, we categorized the public’s compliance behaviour with public health policies based on the results of factor analysis. Subsequently, we examined the impact of government trust and professional trust on compliance behaviour with public health policies by constructing a structural equation model.

**Results:**

Based on the results of factor analysis, we classified public adherence to public health policies into protective compliance and restrictive compliance. Results from the structural equation model show a positive correlation between the public’s trust in the government and both protective and restrictive compliance, with a stronger influence on protective compliance. Government trust also exerts a positive impact on restrictive compliance behaviour through professional trust. Additionally, the study indicates a significant positive correlation between the public’s professional trust and restrictive compliance, while it does not significantly affect protective compliance. Moreover, the public from rural areas demonstrates a greater willingness to adhere to both types of public health policies. Married individuals exhibit a stronger inclination toward protective compliance, while females show a stronger tendency toward restrictive compliance.

**Conclusion:**

The study revealed a significant positive impact of government trust and professional trust on compliance behaviour with public health policies during the COVID-19 pandemic, refuting any negative correlation between government trust and compliance behaviour. Normative motivations for compliance behaviour had a substantial impact on adherence. These findings offer valuable insights for future public health crisis management and public policy formulation.

## Background

Amid the COVID-19 pandemic, numerous nations’ authorities have instituted and enforced a range of containment measures, encompassing social distancing, travel constraints, and vaccination protocols. In the implementation of these policies, the level of public compliance is one of the key factors influencing their effectiveness [1]. However, stimulating public willingness to comply with the epidemic prevention policies remains a challenging task that governments need to address.

Previous studies have indicated that individuals evaluate the suitability and legitimacy of measures based on scientific, legal, moral, and values-based considerations [2]. Research on individual compliance behaviour has categorized motivations into calculative and normative motives. Calculative motives encompass the individual’s inclination to comply after considering the costs and benefits, drawing from theories such as deterrence theory, protection motivation theory, and prospect theory [3–6]. Previous research has extensively examined the influence of factors such as risk perception and self-efficacy on compliance behaviour [7–9]. In contrast, normative motives highlight individuals’ internal moral beliefs, values, and social norms, suggesting that these factors drive voluntary adherence to regulations [10, 11]. Studies have widely explored the effects of factors such as trust in the government, professional trust, individual cognition, and cultural context on compliance behaviour [7, 12–16].

Within these, research on the connection between trust in the government under normative motives and compliant behaviour is abundant, yet substantial differences in viewpoints exist. Some studies suggest a positive association between government trust and compliance behaviour, indicating that the public’s trust in the government leads to a greater inclination to support government measures and solutions, as well as a greater willingness to adhere to related policies [12, 13, 17, 18]. Certain studies have found a weak correlation between government trust and compliance behaviour [19, 20]. There are even studies that have discovered a negative association between government trust and compliance behaviour, exemplified by trust paradox perspectives and support paradox perspectives [21, 22].

The aforementioned discrepancies emphasize the intricate correlation between government trust and compliance behaviour. In the context of contemporary society, the confluence of technological advancements and globalization has engendered a persistent stream of novel risks and uncertainties. People are confronted with complex risk challenges such as environmental pollution, technological disasters, and global climate change, necessitating joint efforts from society, governments, and individuals to address various issues within the realm of risk society [23]. Therefore, studying public compliance behaviour and its motivations is paramount. Our study seeks to investigate the relationship between government trust, professional trust, and compliance behaviour, employing a structural equation model for analysis. This model is commonly utilized to ascertain causal relationships between variables, encompassing both direct and indirect influences. Its strengths lie in evaluating the intricate interconnections among observed variables and testing complex theoretical models.

## Theoretical background

### Compliant behaviour and its motives

Previous research on individual compliance behaviour has primarily been conducted from the perspectives of calculative motives and normative motives. Calculative motives encompass the individual’s motivation to comply after weighing the costs and benefits [24], including deterrence theory, protection motivation theory, and prospect theory. Deterrence theory posits that strict policies and enforcement will enhance the public’s motivation to comply with the policies [6]. The protection motivation theory emphasizes that individual decisions are driven by their perceptions of risk and self-efficacy [4, 5, 25], such as adherence to public health policies for the sake of safeguarding personal interests [26]. Prospect theory divides the decision-making process into two critical stages: an early editing phase that involves a preliminary analysis of the provided prospects, often leading to simplified presentations of these prospects. This is followed by an evaluation phase that assesses the edited prospects, selecting those with the highest value [3].

Differing from calculative motives, normative motives emphasize that individuals’ internal moral beliefs, values, and social norms drive them to voluntarily adhere to regulations. These may include an acknowledgment of social responsibility, concern for the health of others, trust in the government, policy legitimacy, and policy fairness, among other intrinsic values [10, 11]. During the COVID-19 pandemic, research has found that normative motives are the primary driving force behind compliance behaviour [7–9, 17, 27]. Studies have also revealed that government trust and individual responsibility are the primary influencing factors for public compliance behaviour among young people in China during the COVID-19 pandemic [28]. In the context of a pandemic, as the majority of public health policies are issued by the government, government trust becomes a key factor motivating compliance behaviour.

### Government trust and individual compliance behaviour

Government trust encompasses confidence in the government’s consistency in words and actions and its commitment fulfillment [12]. Generally, those who trust the government are more likely to support government measures and solutions and are more willing to comply with related policies [13]. Conversely, low government trust may result in the public’s reluctance to follow government decisions, leading the government to implement mandatory measures to enforce regulations, thus increasing the complexity and cost of governance [29]. Research has shown this to be the case during the COVID-19 pandemic as well. Studies have found that in regions with high government trust in Italy, the public responded actively to government calls, reducing their outings during the pandemic [17]. Other studies have indicated a negative correlation between government trust and the number of COVID-19 cases and mortality rates [30]. Furthermore, there is a mutually reinforcing relationship between the public’s trust in the government and the effectiveness of policies. An increase in government trust can enhance the management of the pandemic, and positive pandemic management outcomes not only restore damaged government trust but also create spillover effects, further increasing public trust in the government. This mutually reinforcing relationship is crucial for establishing a stable and sustainable pandemic management mechanism [31, 32].

However, not all studies consistently demonstrate a positive correlation between government trust and compliance behaviour. Some studies have found a weak association between government trust and compliance behaviour [19, 20]. Jørgensen et al.‘s (2020) research found that institutional trust had a relatively limited promoting effect on compliance behaviour during the first wave of the COVID-19 pandemic [19]. A study conducted in the Netherlands also indicated that higher levels of government trust were only positively related to the willingness to accept vaccination and unrelated to the willingness to adopt additional hygiene measures [25]. Some studies have even discovered a negative correlation between government trust and compliance behaviour. These perspectives are known as the “trust paradox” and the “support paradox.” The trust paradox perspective suggests that confidence in governmental institutions can alleviate the pressures associated with a pandemic. Consequently, the higher the public’s confidence in governmental institutions, the more they may perceive no need to take personal action to avoid infection and restrict the spread of the virus [22]. The fundamental assumption of the trust paradox perspective is that the COVID-19 pandemic is psychologically perceived as a stressor, and trust serves as a psychological defense mechanism, providing a sense of security and stability during uncertain times. Consequently, if the public has confidence in the government’s capabilities and decisions in managing the pandemic, they may be more inclined to believe that the measures taken by the government are sufficient to control the outbreak, thereby reducing the need for individuals to take additional protective measures [21, 33, 34].

Furthermore, the support paradox perspective posits that when the public perceives the government’s effective performance in managing the pandemic, their perception of risk may weaken. Consequently, they might underestimate the severity of the pandemic and their own risk, leading to a relaxation of the importance placed on personal protective measures and a decrease in the willingness to comply with regulations [21]. The support paradox perspective is based on the “trust, confidence, and cooperation (TCC)” model in the field of risk management, which suggests that when confidence is excessive or blind, people may ignore or underestimate potential risks because they excessively rely on a specific responsible party or institution. In such circumstances, higher confidence may lead to a decrease in risk perception. Conversely, when people lack confidence in the relevant parties, they may become more vigilant and sensitive, thus tending to adopt a cautious attitude toward risks. In this scenario, lower trust can lead to an increase in risk perception [21, 23]. In the most severely affected regions in Italy, the effective performance of the regional systems during the pandemic enhanced people’s sense of security, thereby reducing their willingness to comply with restrictive measures, giving rise to the “support paradox” [21].

### The role of professional trust

The aforementioned studies underscore the significant relationship between government trust and adherence to policy compliance. Additionally, research has indicated that public trust in professionals serves as a crucial predictive factor for policy compliance, particularly in the context of public health compliance behaviours, given the intricate and specialized nature of knowledge related to pandemics and infectious diseases [27]. For instance, after previous events such as the avian flu and the 2009 H1N1 influenza pandemic, public trust in professionals emerged as a novel predictive indicator for assessing the adherence to official recommendations concerning protective measures [7]. During the 2009 H1N1 period, following the French public health department’s calls for practices like frequent handwashing and mask-wearing, more than a third of the public reported an increased frequency of handwashing as a preventive measure [14]. A higher level of professional trust among the public corresponds to a greater willingness to seek healthcare assistance and a stronger inclination to adhere to disease prevention measures [35]. Conversely, a crisis of trust between the public and health authorities can have adverse effects, as individuals who harbor distrust in healthcare institutions may not comply with official guidance regarding protective measures [7, 36]. Professional trust encompasses reliance on others’ competence, specialized knowledge, and judgment [37]. Moreover, some research suggests that professional trust acts as an intermediary variable between trust in the government and the adoption of personal protective measures by respondents. In cases where public trust in the government is low, professional trust motivates them to implement personal protective measures, ultimately resulting in satisfactory outcomes [26].

The research also points out that the public’s professional trust is influenced by a combination of various factors, among which trust in the government significantly and strongly affects professional trust [26]. Individuals with a positive and open-minded approach are more inclined to trust public health experts [38]. It is important to note that the public’s interpretation and understanding of pandemic information based on their own knowledge reservoirs can lead to a loss of confidence in health officials [39].

## Research framework and hypotheses

Based on the literature reviewed above, we constructed a model of public health policy compliance behaviour incorporating government trust and professional trust. Public health policy compliance can manifest in various ways as different countries provided different recommendations to their populations during public health crises [40]. For the COVID-19 pandemic, common measures adopted by governments worldwide include wearing masks, hand hygiene, maintaining social distancing in public places, and vaccination. Some governments tended to implement additional measures to protect their populations as their understanding of the crisis deepened [40]. For instance, in order to identify infected individuals, China also implemented measures such as presenting nucleic acid test certificates, health codes and travel card, and refraining from going out during lockdowns. Numerous measures were implemented by various countries during the pandemic, and for the sake of simplicity in the model, we merged multiple types of measures. Some researchers have categorized public health policies into protective compliance and restrictive compliance. Drawing on previous research [28], we similarly divided the public health policies enacted by the Chinese government into protective compliance and restrictive compliance.

Firstly, previous research indicates that during the COVID-19 pandemic, public trust in the government has a significant impact on their adherence to public health measures. Some studies have found that the public’s trust in the government leads to a greater inclination to support and comply with government measures and solutions [1, 12, 13, 17], while lower levels of government trust may result in the public’s reluctance to comply with government decisions, thereby forcing the government to resort to more coercive management measures [29]. Conversely, some scholars have proposed the trust paradox and support paradox viewpoints. The trust paradox viewpoint suggests that the level of public trust in government institutions is negatively correlated with their adherence to public health policies [22]. The support paradox viewpoint posits that during the COVID-19 pandemic, the public’s satisfaction with the government’s performance is negatively correlated with their willingness to adhere to regulations [21]. Therefore, this study presents competing hypotheses regarding the relationship between government trust and adherence to public health policies.

### H1.1

During public health crises, public trust in the government is positively correlated with compliance behaviour (protective compliance a; restrictive compliance b).

### H1.2

During public health crises, public trust in the government is negatively correlated with compliance behaviour (protective compliance a; restrictive compliance b).

In addition to trust in the government making it easier for people to accept existing regulations and restrictions, the public’s professional trust is also closely related to their compliance behaviour [14, 27]. The stronger the public’s professional trust, the stronger their willingness to seek healthcare assistance and adhere to disease prevention measures [14, 35]. Conversely, a trust crisis between the public and health authorities may have adverse effects on public adherence to recommendations [36], as those who do not trust healthcare institutions may not follow official protective measures [7]. Therefore, we propose hypotheses H2.1 and H2.2.

### H2.1

During the public health crisis, the public’s professional trust is positively correlated with protective compliance behaviour.

### H2.2

During the public health crisis, the public’s professional trust is positively correlated with restrictive compliance behaviour.

Previous research has also indicated that professional trust plays a mediating role between government trust and respondents’ adoption of personal protective measures. When the public has lower levels of trust in the government, professional trust prompts them to take individual protective measures, ultimately yielding satisfactory results. Therefore, we propose hypotheses H3.1 and H3.2.

### H3.1

Government trust has a positive effect on protective compliance through professional trust.

### H3.2

Government trust has a positive effect on restrictive compliance through professional trust.

**Fig. 1:**
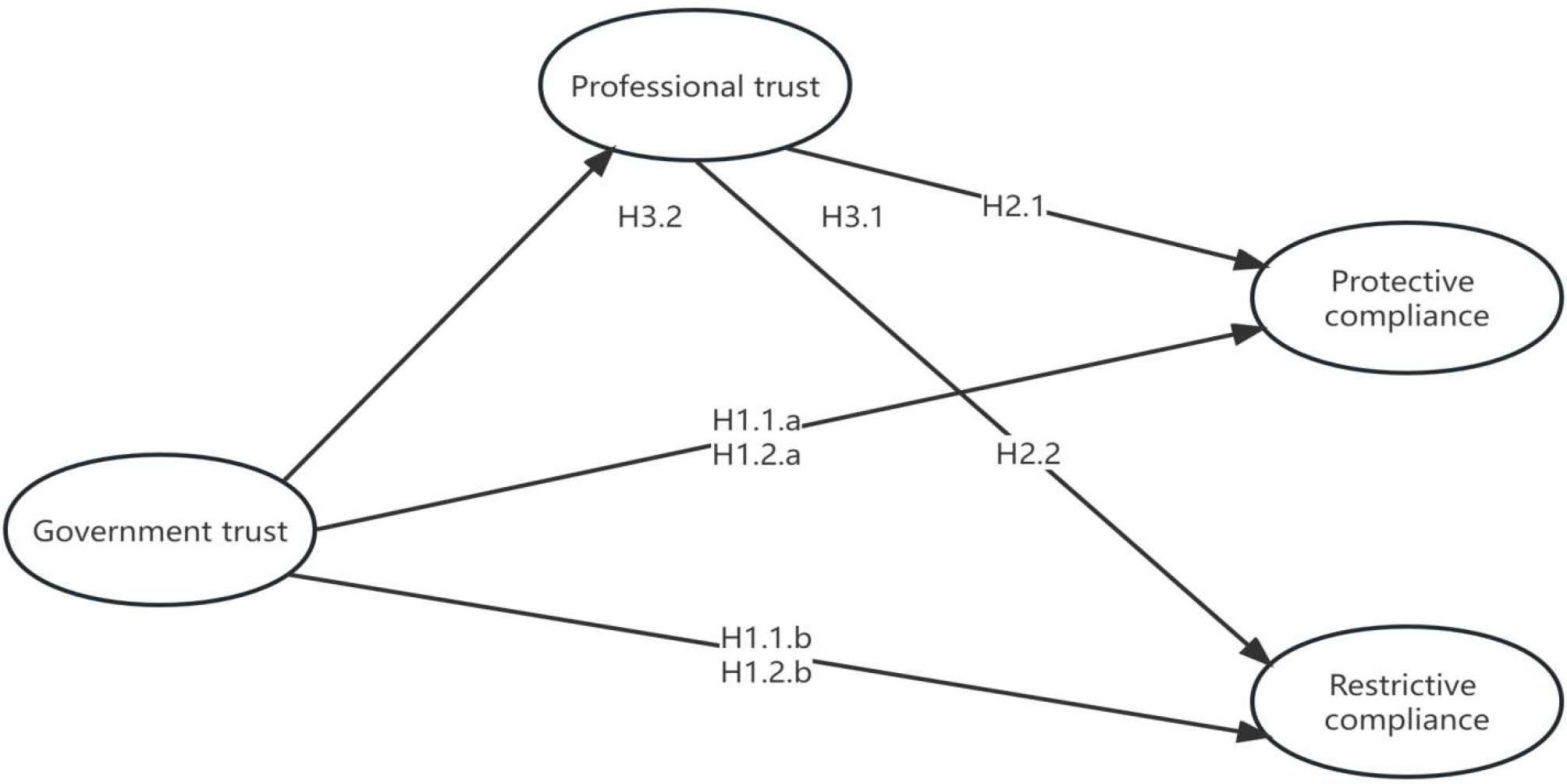
The hypothesized theoretical model with a structural equation model

Figure 1 illustrates the theoretical framework and hypotheses of this study.

## Materials and methods

### Data collection

The questionnaire for this study was distributed from mid-November to late December 2022. Due to the restrictions of the home isolation policy, from mid-November to early December, we only distributed the questionnaire to university students through online platforms such as email and social media. In mid-December 2022, with the lifting of the home isolation policy, we used offline methods to distribute the questionnaire to on-campus students. A total of 1436 university students completed the questionnaire, distributed across 25 administrative regions. To ensure that participants would not be coerced into completing the questionnaire, we included an anonymous statement in the first part of the questionnaire. After completing the data collection, we excluded invalid questionnaires that were duplicates or incomplete, ultimately obtaining 1395 valid questionnaires.

### Statistical methods

After the completion of data collection, we conducted statistical analysis using STATA 15.1 software. Firstly, descriptive analysis of the data was performed. Subsequently, we conducted factor analysis and constructed a structural equation model to delve into the impact of public trust in the government and professional trust on their compliance with public health policies.

### Standard protocol approvals, registrations, and participants consent

Standard protocol approvals, registrations, and participants consent Informed consent was obtained from all the participants in the study. The study was approved by the Academic Ethics Committee of School of Politics Science and Public Administration, Guangxi Minzu University. The present study was conducted in accordance with the principles of the 2013 Declaration of Helsinki.

### Variables and measurements

#### Compliance behaviour

The public’s compliance with public health policies is the main outcome variable of the study. Compliance with public health policies can take various forms, as different countries have provided their populations with different recommendations during public health crises [40]. During the COVID-19 pandemic, wearing masks, handwashing, maintaining social distance in public places, and vaccination have been commonly adopted policies by governments worldwide. Following a deeper understanding of the crisis, some governments inclined towards implementing additional measures to protect the public [40]. For instance, in China, measures such as presenting nucleic acid test certificates, health codes and travel card, and refraining from going outside during lockdown periods were adopted to identify infected individuals. To assess the public’s adherence to public health policies during the COVID-19 pandemic, we employed a Likert five-point scale to measure respondents’ compliance with seven official recommendations (wearing masks, handwashing, maintaining social distance in public places, vaccination, presenting nucleic acid test certificates, presenting health codes and travel card, and refraining from going outside during lockdown periods) (Cronbach’s alpha = 0.81). Participants were required to select a numerical value ranging from 1 to 5 based on statements, with 1 indicating “never complied” and 5 indicating “always complied.” Higher scores indicate a higher level of compliance with public health policies among the respondents.

According to the results of the factor analysis of the seven compliance behaviours, after orthogonal rotation, two factors with eigenvalues greater than 1 were obtained, namely 2.188 and 1.636. The variance explained by the two factors was 0.6614 and 0.4945, respectively. Based on the results of the factor analysis and drawing on the research by Huang (et al., 2023) [28], we divided the public’s compliance with public health policies into two main factors (see Table 1). Factor 1 was named “Restrictive Compliance” (Cronbach’s alpha = 0.79), including vaccination, presenting nucleic acid test certificates, presenting health codes and travel card, and refraining from going outside during lockdown periods, with factor loadings of 0.6121, 0.7941, 0.8384, and 0.5637, respectively. Factor 2 was named “Protective Compliance” (Cronbach’s alpha = 0.82), including wearing masks, handwashing, and maintaining social distance in public places, with factor loadings of 0.6692, 0.6819, and 0.7102, respectively.

**Table 1:** Factor analysis result

Compliance behavior	Variable	Factor1	Factor2	Uniqueness
Protective compliance	Wear a mask	0.273	0.669	0.478
	Wash hands	0.245	0.682	0.475
	Maintain social distancing	0.169	0.710	0.467
Restrictive compliance	Get vaccinated	0.612	0.209	0.582
	Nucleic acid test	0.794	0.234	0.314
	Health code	0.838	0.160	0.272
	Stay indoors	0.564	0.3081	0.587
Trust	Variable	Factor1		Uniqueness
Government trust	Central government	0.78		0.392
	Local government	0.78		0.392
Professional trust	Medical personnel	0.845		0.286
	Medical institution	0.845		0.286

#### Government trust

Government trust is a key independent variable in this study. To measure respondents’ levels of trust in the government, we asked the following two questions: “Currently, how satisfied are you with the central government?” and “Currently, how satisfied are you with the local government in your district/county?” Participants were required to select a value within the range of 1 to 5, where 1 represented “very dissatisfied” and 5 represented “very satisfied.” Higher scores indicated a higher level of trust in the government among the respondents (Cronbach’s alpha = 0.83).In our study, the government trust variable was constructed through exploratory factor analysis, examining the questions “Currently, how satisfied are you with the central government?” and “Currently, how satisfied are you with the local district/county government?” After orthogonal rotation, a factor with an eigenvalue of 1.217 and a variance explained of 1.203 was obtained, with a factor loading of 0.78 (see Table 1).

#### Professional trust

Professional trust is also a crucial variable in this study. To measure respondents’ levels of professional trust, we asked the following two questions: “How much do you trust healthcare professionals?” and “How much do you trust healthcare institutions?” Participants were required to select a value between 1 and 5, where 1 represented “strongly distrust” and 5 represented “strongly trust.” Higher scores indicated a higher level of professional trust among the respondents (Cronbach’s alpha = 0.88).The professional trust variable was also constructed through exploratory factor analysis, examining the questions “How much do you trust healthcare professionals?” and “How much do you trust healthcare institutions?” After orthogonal rotation, a factor with an eigenvalue of 1.429 and a variance explained of 1.128 was obtained, with a factor loading of 0.845 (see Table 1).

### Demographic variables

Previous research has shown that age, gender, and marital status have important effects on individual compliance behaviour [41–43]. In our study, we used four demographic variables: the respondent’s gender (0 = male, 1 = female); the respondent’s marital status (0 = single, 1 = married); the respondent’s political affiliation (0 = Chinese Communist Party member, 1 = non-Chinese Communist Party member); the respondent’s household registration (0 = rural household registration, 1 = urban household registration).

## Result

### Demographic characteristics

Table 2 displays the basic characteristics of the control variables. Among the 1395 samples, male citizens accounted for 22.58%, while female citizens accounted for 77.42%; Citizens under the age of 24 account for 93.32%, citizens between 24 and 35 years old account for 5.52%, and citizens over the age of 35 account for 1.15%.citizens with urban household registration accounted for 37.92%, while those with rural household registration accounted for 62.08%. Citizens with membership in the Chinese Communist Party accounted for 7.60% of the total sample, while citizens without membership accounted for 92.40%.Single citizens account for 94.77%, married citizens account for 5.23%. Citizens with education below college level account for 9.46%, while citizens with college education or above account for 90.54%.

**Table 2:** Descriptive statistics of the demographic characteristics of the study population (*n* = 1395)

Variable	Category	n (%)
Gender	Male	315(22.58)
	Female	1080(77.42)
Age	< 24	1132(93.32)
	24–35	67(5.52)
	> 35	14(1.15)
Marital status	Single	1322(94.77)
	Married	73(5.23)
Education	below undergraduate level	132(9.46)
	Bachelor’s degree and above	1263(90.54)
Political landscape	CCP member	106(7.60)
	Non-CCP member	1289(92.40)
Household registration	urban	529(37.92)
	rural	866(62.08)

### Descriptive statistics and correlation analysis of study variables

Table 3 presents the descriptive statistics and correlation analysis of the study variables. Among the seven compliance behaviours, “Providing health codes and travel card” had the highest average score (4.85), followed by “Providing nucleic acid test certificates” (4.80), “Vaccination” (4.77), “Compliance with not going out during lockdown periods” (4.74), “Wearing masks” (4.42), “Handwashing” (4.40), and the lowest average score was for “Maintaining social distance in public places” (3.90). Regarding government trust, the average score for the central government was 4.10, slightly higher than that for the district/county government (3.83). As for professional trust, the average score for trust in healthcare professionals (4.07) did not differ much from the average score for trust in healthcare institutions (4.03).

During the COVID-19 pandemic, the average scores for the four restrictive compliance behaviours, “Presenting health codes and travel card,” “Presenting nucleic acid test certificates,” “Vaccination,” and “Compliance with not going out during lockdown periods,” were much higher than the average scores for protective compliance (handwashing, wearing masks, maintaining social distance in public places). This result is consistent with our research expectations. This is because during the COVID-19 pandemic, the Chinese government implemented strict restrictive public health policies to ensure the safety of public mobility, and non-compliance with these policies would result in direct consequences of restricted mobility.

During the COVID-19 pandemic, we observed a series of behavioural differences related to public characteristics, which to some extent reflected different responses to prevention and control measures (see Table 3). We found that individuals with rural household registration were more willing to wear masks (*p* = 0.025), handwashing (*p* = 0.016), present nucleic acid test certificates (*p* < 0.001), present health codes and travel card (*p* = 0.003) and comply with not going out during lockdown periods (*p* = 0.001). Citizens with membership in the Chinese Communist Party were more willing to get vaccinated (*p* = 0.036). Additionally, males showed a higher willingness to maintain social distance in public places (*p* = 0.018), while females were more willing to present nucleic acid test certificates (*p* = 0.013) and health codes and travel card (*p* = 0.004). Married individuals paid more attention to hand hygiene during the pandemic (*p* = 0.034).

**Table 3:** Descriptive statistics and correlation analysis of study variables (*n* = 1395)

Compliance behaviour	Mean ± SD	Gender	Household registration	Political membership	Marital status
Wear a mask	4.42 ± 0.73	-0.04	-0.06*	0.02	0.05
Handwashing	4.40 ± 0.73	0.13	-0.06*	0.02	0.06*
Maintain social distancing	3.90 ± 0.97	0.06*	-0.03	-0.01	0.04
Vaccinate	4.77 ± 0.56	-0.05	-0.04	0.06*	0.02
NAT certificate	4.80 ± 0.49	-0.07*	-0.09***	0.04	0.02
Health code	4.85 ± 0.41	-0.08**	-0.08**	0.03	0.05
Stay home	4.74 ± 0.55	-0.04	-0.09**	-0.01	-0.02
Protective compliance	4.24 ± 0.68	0.02	-0.06*	0.01	0.06*
Restrictive compliance	4.79 ± 0.41	-0.01**	-0.09***	0.02	0.02
Government trust	3.96 ± 0.83	0.04	-0.14***	-0.01	-0.05
Professional trust	4.05 ± 0.71	0.001	-0.02	-0.02	-0.05

*Note* *, **and *** indicate *p* < 0.05 *p* < 0.01 and *p* < 0.001, respectively

### The results of SEM

**Fig. 2:**
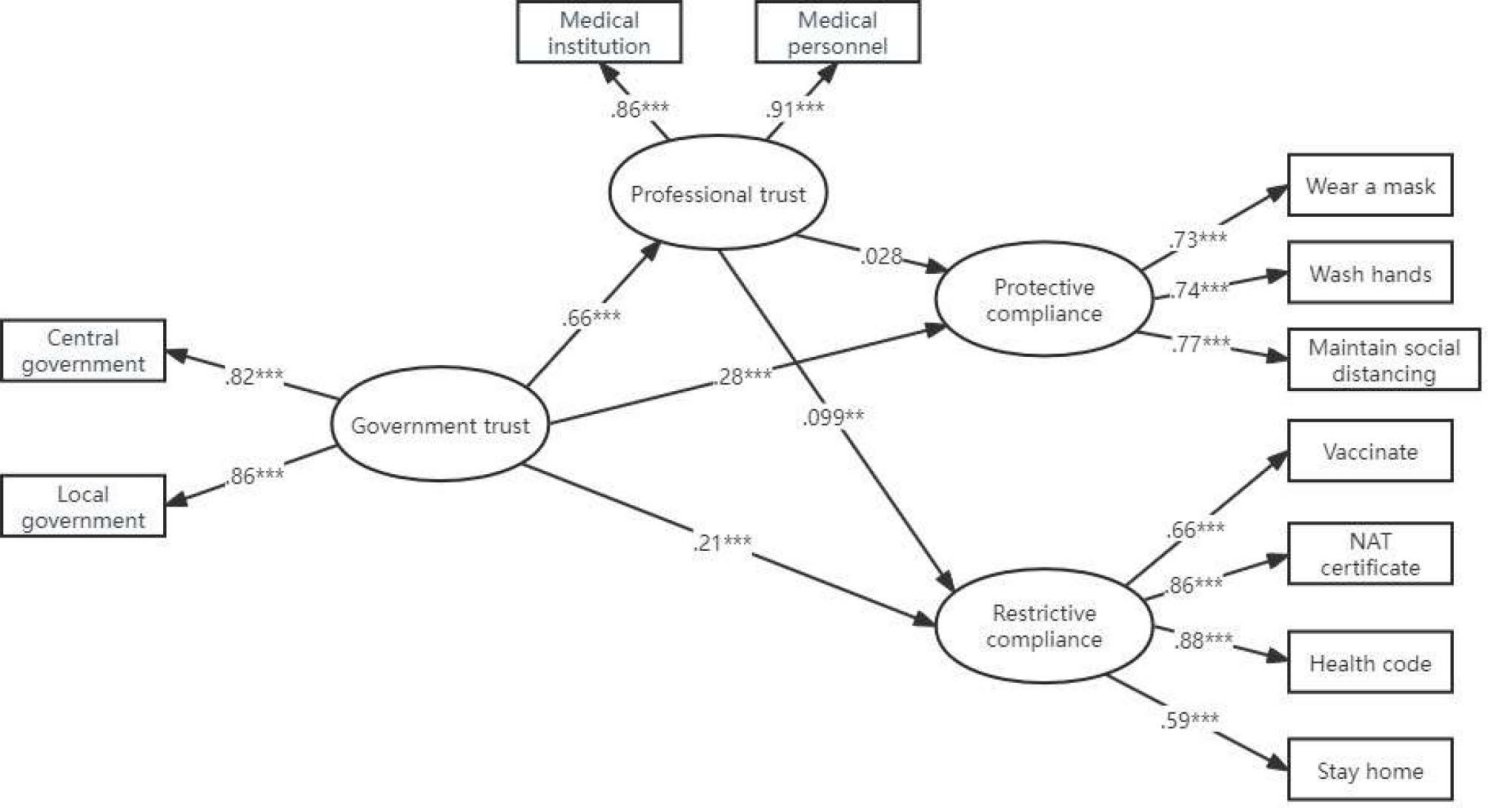
Mediation model with SEM. *Note* Control variables (gender, age, education, marital status, political landscape and household registration) were included in this model but not presented in this figure

In order to further explore the impact of government trust and professional trust on public compliance with public health policies, we constructed a structural equation model (see Fig. 2). The model showed statistical significance (χ² = 461.70, df = 93, *p*<0.001) and exhibited acceptable fit indices (CFI = 0.940, TLI = 0.921, RMSEA = 0.057, SRMR = 0.077).

The structural equation model results indicate that public trust in the government significantly influences several factors. Firstly, public trust in the government is significantly positively correlated with protective compliance (a = 0.28, *p*<0.001; see Table 4). Hypothesis H1.1.a is supported, while its competing hypothesis H1.2.a is not supported. Secondly, public trust in the government is also significantly positively correlated with restrictive compliance (a = 0.21, *p*<0.001; see Table 4). Hypothesis H1.1.b is supported, while its competing hypothesis H1.2.b is not supported. Additionally, government trust is significantly positively correlated with professional trust (a = 0.66, *p*<0.001; see Table 4).

**Table 4:** Direct and indirect effects analysis(*n* = 1395)

Direct and Indirect Effects	Estimate effects(SE)
Government trust→Protective compliance(H1.1.a、H1.2.a)	0.28***(0.049)
Government trust→Restrictive compliance(H1.1.b、H1.2.b)	0.21***(0.049)
Professional trust→Protective compliance(H2.1)	0.028(0.05)
Professional trust→Restrictive compliance(H2.2)	0.099**(0.047)
Government trust→Professional trust	0.66***(0.02)
Indirect effect [95% bootstrap] Government trust→Protective compliance(H3.1)	0.013[-0.033, 0.06](0.024)
Indirect effect [95% bootstrap] Government trust→Restrictive compliance(H3.2)	0.032 [0.002, 0.062](0.015)
Age→Protective compliance	0.067(0.035)
Age→Restrictive compliance	-0.022(0.033)
Gender→Protective compliance	0.03(0.031)
Gender→Restrictive compliance	-0.07**(0.029)
Household registration→Protective compliance	-0.038(0.032)
Household registration→Restrictive compliance	-0.05(0.03)
Political landscape→Protective compliance	0.009(0.031)
Political landscape→Restrictive compliance	0.036(0.03)
Educational level→Protective compliance	-0.003(0.031)
Educational level→Restrictive compliance	0.074**(0.029)
Marital status→Protective compliance	0.057(0.035)
Marital status→Restrictive compliance	0.06(0.033)

Note *p < 0.05,**p < 0.01,***p < 0.001

We also found that public professional trust is significantly positively correlated with restrictive compliance (a = 0.099, *p*<0.001; see Table 4), confirming hypothesis H2.b. However, we did not find a significant effect of public professional trust on protective compliance (a = 0.028, *p* = 0.57; see Table 4), and hypothesis H2.a was not supported.

Furthermore, we also found that government trust has a significant positive effect on restrictive compliance through professional trust (indirect effect = 0.032, 95% confidence interval [0.002, 0.062]; see Table 4), confirming hypothesis H3.b. However, government trust did not have a significant positive effect on protective compliance through professional trust (indirect effect = 0.013, 95% confidence interval [-0.033, 0.06]; see Table 4), and hypothesis H3.a was not supported. These findings emphasize the shaping role of government trust during the COVID-19 pandemic on professional trust, protective compliance, and restrictive compliance, as well as the significant influence of professional trust on public restrictive compliance behaviour.

In terms of controlling variables, the results of the structural equation model show that there is no significant correlation between respondents’ age and protective compliance (*p* = 0.06) or restrictive compliance (*p* = 0.49); respondents’ gender is not significantly correlated with protective compliance (*p* = 0.33), and it is significantly negatively correlated with restrictive compliance (β=-0.07, *p* = 0.017), although this association is weak; respondents’ household registration is not significantly correlated with protective compliance (*p* = 0.24) or restrictive compliance (*p* = 0.09); respondents’ political affiliation is not significantly correlated with protective compliance (*p* = 0.76) or restrictive compliance (*p* = 0.22); respondents’ educational level is not significantly correlated with protective compliance (*p* = 0.93), and it is significantly negatively correlated with restrictive compliance (β = 0.07, *p* = 0.012), although this association is weak; respondents’ marital status is not significantly correlated with protective compliance (*p* = 0.1) or restrictive compliance (*p* = 0.07).

## Discussion

During the COVID-19 pandemic, compliance with epidemic prevention measures is not only an individual’s autonomous choice but also a responsible act and an expression of care for the entire society. Adhering to public health policies, such as wearing masks, maintaining social distance, and getting vaccinated, can effectively slow the spread of the virus, reduce the risk of infection, and protect vulnerable groups and healthcare workers from the virus. Therefore, during the pandemic, the public’s compliance behaviour is a crucial element in overcoming the virus and maintaining social security. Although existing research has indicated that public trust in the government and professional trust are important factors influencing compliance behaviour, there are still some controversies regarding this relationship. Hence, this study constructed a structural equation model to examine the impact of government trust and professional trust under normative motivation on compliance with public health policies during the COVID-19 pandemic.

### Vaccination: self-protection or external pressure?

The research findings indicate that, following factor analysis, vaccination was classified as a form of restrictive compliance behaviour. This contradicts previous research, which regarded vaccination during pandemics as a proactive self-protective measure taken by the public [28]. We suggest two explanations for this phenomenon. Firstly, during the COVID-19 pandemic, many countries have implemented restrictive policies targeting unvaccinated individuals, leading to various limitations on their travel, education, employment, and accommodation in hotels [44, 45]. Secondly, widespread vaccine hesitancy regarding the safety of COVID-19 vaccines exists globally [46–50]. This suggests that individual vaccination may be more driven by meeting external demands rather than purely self-protective motives.

### Government trust and compliance behavior

The results of the structural equation model indicate that public trust in the government significantly affects both protective compliance and restrictive compliance, with a stronger impact on protective compliance. Our study initially confirmed the positive association between government trust and compliance behaviour [13, 17, 18], not supporting a weak correlation or a negative association between government trust and compliance behaviour. Citizens who trust the government are inclined to perceive government-recommended actions as more beneficial, with fewer obstacles and disadvantages, and as more feasible [51]. Therefore, they are more likely to support government-proposed measures and solutions, including preventive measures, fiscal aid and economic stimuli, as well as epidemic control solutions [12]. Moreover, during the COVID-19 pandemic, if some individuals begin to express satisfaction with and support for the government’s measures, this positive sentiment could spread to their social circles, influencing more people to adhere to the government’s epidemic guidance, as they perceive the government as trustworthy and taking appropriate measures to address the crisis. This phenomenon can be described as a cascade of confidence, wherein confidence spreads from one individual or group to another, creating a chain reaction [22].

### Government trust and professional trust

The study results further indicate that the public’s trust in the government also makes them more likely to trust professional healthcare institutions and medical personnel. This suggests that the government plays a crucial role in maintaining the independence and credibility of healthcare institutions and medical personnel [26]. When formulating public health risk policies and governance decisions, the government often relies on expert systems for decision-making and risk assessment. In the eyes of the public, both expert systems and the government are perceived as “rational-legitimate” authorities [52]. This leads to the common association of trust in expert systems with trust in the government, forming a shared basis of trust in this context [52].

### Professional trust is significantly positively correlated with restrictive compliance

Our study also confirms the positive influence of professional trust on compliance with public health policies, consistent with previous research [7, 26, 35, 36, 38, 41, 53]. When the public perceives the medical knowledge of healthcare institutions and medical staff as trustworthy, they are more likely to actively follow their recommendations [7]. Furthermore, we observed the mediating effect of professional trust, where government trust positively influences compliance with restrictive measures through professional trust. During a crisis, professional trust serves as a complementary mechanism, enhancing the likelihood of the government’s ability to encourage individuals to adopt personal protective measures [26]. When both government trust and professional trust are strong, the pandemic can be better controlled [26].

### Professional trust has no significant impact on protective compliance

However, this study found that the public’s trust in professional healthcare institutions and medical staff tends to lean towards compliance with restrictive measures rather than protective measures. The results of the structural equation model also demonstrate that professional trust, as an intermediary for government trust, only affects compliance with restrictive measures and does not impact the effect of protective compliance. One possible explanation is that professional healthcare institutions and medical staff play a critical role in implementing restrictive measures, such as vaccine administration, providing nucleic acid test certificates, displaying health codes and travel card, and adhering to home quarantine during lockdown periods. In contrast, protective measures rely more on the voluntary compliance of the public, as these measures require spontaneous actions from the public, with relatively less involvement from professional healthcare institutions and medical staff. In China, the public’s vaccine administration and nucleic acid testing are primarily managed by professional healthcare institutions and medical staff, while the management of health codes and travel cards, as well as home quarantine during lockdowns, is a joint responsibility of the government and professional healthcare institutions [54–57]. Therefore, the public’s trust in professional healthcare institutions and medical staff tends to be more aligned with compliance with restrictive measures.

### Analysis of compliance behavior differences among different demographic groups

We also analyzed the behavioural differences among different groups during the COVID-19 pandemic. We found that the public with rural household registration displayed a higher willingness to comply with public health policies, consistent with existing research findings [28]. This could be attributed to the relatively poor economic conditions in rural areas, leading them to rely more on the healthcare and sanitation resources provided by the government to ensure access to necessary medical services. Additionally, some rural communities may possess strong traditional cultural values that prompt the public to participate more actively in social activities and respond to government calls, such as adhering to public health policies. Finally, we observed that women exhibited a stronger inclination toward restrictive compliance, which could be due to their better understanding of COVID-19 and their relatively lower likelihood of engaging in risky behaviours [15, 58].

### The impact of research results on public health policies

To further enhance public compliance with public health policies during public health crises, it is essential to consolidate and enhance trust in the government. Firstly, there should be continued efforts to strengthen transparency and communication. Timely, transparent, and accurate dissemination of information to the public during public health crises forms the foundation of trust. Secondly, it is necessary to flexibly address the diverse needs of citizens. Different groups and regions may have varying needs and challenges during public health crises. By considering the diversity of public needs and tailoring policies accordingly, the government can better gain public understanding and support, thus enhancing trust. Additionally, to further improve public compliance with public health policies during public health crises, it is crucial to consolidate and enhance trust in medical professionalism. Healthcare institutions and the government should actively communicate authentic information about the pandemic, explain the scientific basis behind health policies, and share opinions and advice from medical professionals. Clear communication enables the public to better understand the urgency and necessity of healthcare decisions, thereby increasing trust in the healthcare system. Finally, ensuring that public health policies and practices adhere to international standards and professional guidelines can enhance public trust in professionalism. This includes providing scientific explanations about vaccination, treatment methods, preventive measures, and timely updates on medical information to adapt to the evolving pandemic situation.

### Limitations and future directions of the study

Nevertheless, it is important to acknowledge some limitations of this study. Firstly, the study employed a convenience sampling method, targeting university students. Therefore, the representativeness of the sample might be somewhat limited, making it difficult to generalize to the entire Chinese population. Secondly, the study relied on self-reported data from the respondents, which could lead to memory biases and social desirability biases. Participants might exaggerate or underestimate their compliance with public health policies or be influenced by social expectations, leading to inaccuracies in the data. In conclusion, although this study provides useful information about the compliance behaviour and trust levels of the Chinese public during the COVID-19 pandemic, the aforementioned limitations need to be considered when interpreting and generalizing the research findings. As of January 8, 2023, China has lifted the preventive and control measures for COVID-19, and COVID-19 is no longer classified as a class A infectious disease under quarantine management. Therefore, future research could further expand the sample size, enhance the representativeness of the sample, and consider more potential factors to study in depth the relationship between public compliance behaviour and trust during health crises.

## Conclusion

This study delved into the influence of government trust and professional trust on public compliance with public health policies under normative motivations, and categorized public compliance behaviour into protective compliance and restrictive compliance, making the research conclusions more specific. The study found that during the COVID-19 pandemic, both government trust and professional trust significantly impacted the public’s adherence to public health measures. To enhance public compliance with COVID-19 preventive measures, it is important to reinforce the public’s sense of moral obligation and establish trust in government authorities to encourage active participation [59]. Governments can reduce the negative impact of information conflicts on government trust by providing fair, transparent, authentic, and two-way communication, as well as by building and popularizing e-governance [60–63]. During crises, it is also essential to emphasize the role of crisis interventions in restoring government trust [32], thereby maintaining the credibility of professional medical institutions and healthcare personnel to better understand and address public health challenges. These findings are not only significant for the current health crisis but also provide valuable insights for future health crises and government policy-making.

## Data Availability

If you need us to provide data, please contact the correspondence author.

## Abbreviations

CCP: China Communist Party
